# Supplementary material for: Picosecond to millisecond tracking of a photocatalytic decarboxylation reaction provides direct mechanistic insights
Source: Nat Commun. 2019 Nov 13;10:5152. doi: 10.1038/s41467-019-13154-w (PMC6853971; doi:10.1038/s41467-019-13154-w)
Supplement: Supplementary file 1 — Supplementary Information [file 41467_2019_13154_MOESM1_ESM.pdf]

SUPPLEMENTARY INFORMATION
Picosecond to millisecond tracking of a photocatalytic decarboxylation reaction provides direct mechanistic insights

Bhattacherjee et al.

Supplementary Methods

Experimental setup All femtosecond-to-millisecond time-resolved infrared measurements reported in the main text are carried out at the 'LIFEtime' facility of Rutherford Appleton Laboratory. Details of the apparatus are outlined elsewhere.[1] All femtosecond-to-nanosecond time-resolved electronic measurements and time-resolved infrared experiments (specifically, Supplementary Figure 3) reported in the Supplementary Materials are carried out in the ultrafast laser laboratory at Bristol. The experimental setup at Bristol has been described earlier.[2]

TRIR experiments at LIFEtime make use of an ultraviolet pump beam (256 nm for experiments on PHEN and 375 nm for experiments on Anthracene) and two independent mid-infrared probe beams that can be tuned to different regions (e.g. nitrile stretch and C-C stretch/C-H bend). This wavelength control enables the probing of two different chemical functionalities in the same experiment. Two Yb:KGW ultrafast amplifiers (Light Conversion Pharos - 1030 nm, 15 W, 100 kHz, 260 fs pulses and Pharos SP - same except 6 W, 180 fs pulses) are used to generate the single UV pump and dual IR probe beams as the output of commercial optical parametric amplifiers (Light Conversion, Orpheus HP and Orpheus ONE). The instrument response of the setup is 200 fs. The spectral resolution is 1-2 cm^{-1}.

The probe beams are focused using a 7.5 cm focal-length gold parabolic mirror (50 to 75 μm spot size) on to the sample, overlapped with the focused pump beam (spot size of 120-150 μm diameter). The relative polarization of the pump and probe beams is held at magic angle. The residual pump is blocked and the two probe beams are dispersed in two different spectrometers fitted with 128-element MCT detector arrays (IR Associates). The pump repetition rate is 1 kHz and the probe repetition rate is 100 kHz. The last probe spectrum in the 100-pulse sequence is used for the 'pump-off' measurement. Thus, the absorbance of the sample is measured as $A = -log_{10}\frac{I_T}{I_0}$ and the differential absorbance as $\Delta A(t) = -log_{10}\frac{I_{on}}{I_{off}}$. Here, I_T and I_0 denote the transmitted and incident probe intensities, whereas I_{on} and I_{off} denote the transmitted probe intensities in the presence and absence of the pump pulse. The superior shot-to-shot stability of the Yb:KGW laser systems allows measurement of optical densities down to 10^{-5} OD.

A typical TRIR experiment averages over two seconds (2×10^5 probe laser pulses and 2×10^3 pump laser pulses) and three repeat cycles of (i) 117 picosecond-to-nanosecond time-points (controlled by a two-pass, 8-ns optical delay stage) and (ii) additional timepoints with fixed incremental timesteps of 12 ns (oscillator roundtrip time) and 10 μs (amplifier repetition rate). Such an experiment takes a real runtime of 12 minutes. The infrared spectra are calibrated using the known infrared absorption bands of polystyrene, acetonitrile, acetonitrile-d_3, dichloromethane-d_2, and

carbon dioxide. The pump energy is 80 nJ/pulse at 256 nm and 400 nJ/pulse at 375 nm. Approximately, 0.05 μJ/pulse is used for the mid-IR probes. The sample solution is flowed in a Harrick cell (1.5 mm thick CaF_2 windows separated by 100 μm teflon spacers which set the pathlength) using a peristaltic pump and the Harrick cell is rastered to excite a fresh part of the sample and prevent product accumulation or optical damage. The IR beam path is continually purged with nitrogen to remove ambient CO_2 and water vapor.

TRIR experiments at Bristol employ a single UV-pump and a single mid-IR-probe beam, as described earlier.[2] An ultrafast amplifier (6W, 1 kHz, sub-40 fs pulses at 800 nm, Coherent Legend) is used to drive two optical parametric amplifiers (OPerA-Solo) to generate the UV and mid-IR beams via various nonlinear frequency-mixing schemes of various combinations of the signal, idler, and pump beams. For example, 256 nm is the second harmonic of the sum frequency of the signal with the pump, 375 nm is the fourth harmonic of the signal, and mid-IR wavelengths are produced by the difference frequency of the signal and idler. The pump and probe beams are focused at the sample, which is made to flow through a continually rastered-Harrick cell using a peristaltic pump. The pump beam is routed through a variable optical delay stage (1.5 ns travel length) to control the pump-probe time delays. The infrared beam output is split into two arms for active baseline correction. The transmitted infrared is detected by dispersion on to two separate cryogenically-cooled, MCT arrays (128 element) mounted in spectrometers (Horiba). The instrument response for this setup is 300 fs. These experiments use the same scheme of measuring differential absorbance as described before for the LIFEtime facility.

Time-resolved electronic spectroscopy (TRES) measurements make use of a UV pump (256 or 375 nm) and white-light continuum probe (300-700 nm) generated by focusing 800 nm (< 1 μJ) pulses into a rastered, 1.5 mm thick CaF_2 window. The probe is recollimated using an off-axis parabolic mirror, focussed and overlapped with the pump beam at the sample. The transmitted probe spectrum is dispersed on to a 1064-element array detector (Andor, Oxford Instruments). The impulsive stimulated Raman response of the solvent is used to determine time-zero and the instrument response (150 fs). A typical TRES experiment averages over two seconds (2×10^3 pulses) and two or three repeat cycles of 88 timepoints distributed over femtosecond-to-nanosecond dynamic range. The spectrometer is calibrated against the electronic absorption spectrum of Holmium oxide as a reference.

Sample preparation Phenanthrene (PHEN), anthracene (ANTHRA), 1,4-dicyanobenzene (DCB), cyclohexanecarboxylic acid (CHCA), acetonitrile, and acetonitrile-d_3 are purchased from Sigma Aldrich and used without further purification. UV-VIS steady state absorption spectra (Supplementary Note 2) are measured in a 200 μm Harrick cell in acetonitrile to determine the optimum

concentrations of the various compounds in the reaction mixture needed to ensure selective excitation of the arene. For time-resolved experiments, all solutions are made in deuterated acetonitrile in the case of TRIR and normal acetonitrile in the case of TRES. The total volume of the initial solution is 7 ml, and an equimolar ratio of PHEN (10 mM) and DCB (10 mM) is used. Additionally, TRES studies of PHEN (10 mM) and ANTHRA (10 mM) in acetonitrile are carried out to probe the intrinsic photophysics of the molecules.

All solutions are made in glass vials of 25 ml volume. The quantities of the compounds needed to make the desired concentration are weighed using a digital balance. The requisite solvent volumes are withdrawn using a 10 ml glass syringe. All glassware is cleaned with laboratory-grade acetone and completely dried in an oven before use. We use the starting reactants (i.e. PHEN and DCB or ANTHRA and DCB) at concentrations similar to those reported in organic reactions throughout.[3] The concentration-dependent studies of photoinduced electron transfer are carried out by preparing a 100 mM stock solution of DCB and adding measured amounts to the solution, sequentially, using a 1 ml graduated syringe. Although this marginally dilutes the PHEN concentration in solution, it is confirmed that the procedure does not cause photoexcitation of DCB at the highest concentrations sampled (27 mM) because of the low oscillator strength of DCB at the chosen excitation wavelengths. The concentration ratio of PHEN and CHCA (in NaOH solution) is varied from 1:1 to 1:4 to observe the decarboxylation step.

The solutions are flowed through a Harrick cell using a peristaltic pump with teflon tubings. The components involved in the circulation are thoroughly cleaned between successive scans using the pure solvent (first normal and then deuterated) to prevent contaminations, without wasting expensive deuterated acetonitrile. The open end of the sample vial is fitted with a rubber septum containing piercings to enable the passage of 1/8" teflon tubings for circulation. A nitrogen balloon is used to prevent the entry of ambient air into the sample vial headspace. One notable difference in the conditions of our experiment with those of organic synthesis is that we do not use an argon atmosphere to carry out the reaction, which although found to be critical for maintaining high product yields, is not necessary for a spectroscopic investigation.

Kinetic modeling of the decarboxylation reaction A three-state sequential reaction model[4] is applied to obtain the overall kinetics for the decarboxylation reaction and the lifetime of the carboxyl radical, in particular.

$$A \xrightarrow{k_1} B \xrightarrow{k_2} C$$

Here, A = CHCA anion, B = CHC radical, and C = CO_2. With reference to the reaction scheme shown in Figure 1, we can also write A = $RCOO^-$, B = $RCOO^\bullet$, and C = $R^\bullet$ or equivalently, CO_2. Note that in the transient absorption experiment, the CHCA anion is not directly tracked. However,

from the oxidative step (step 3) of the reaction we can infer that the decay of the CHCA anion must mirror the decay of the PHEN cation, the latter being directly observed in the experiment. The rate constants k_1 and k_2 shown in the equation above are related to the $1/e$ lifetimes of the species A and B as $k_1 = \frac{1}{\tau_1}$ and $k_2 = \frac{1}{\tau_2}$. From the rate equations for this reaction scheme, the time-dependent populations of the various species can be shown to be[4]

$$[A] = [A]_0 \cdot exp\left(\frac{-t}{\tau_1}\right)$$

$$[B] = \frac{\tau_2[A]_0}{\tau_1 - \tau_2} \cdot \left[exp\left(\frac{-t}{\tau_1}\right) - exp\left(\frac{-t}{\tau_2}\right)\right]$$

$$[C] = [A]_0 \cdot \left[1 - \frac{\tau_1}{\tau_1 - \tau_2} \cdot exp\left(\frac{-t}{\tau_1}\right) + \frac{\tau_2}{\tau_1 - \tau_2} \cdot exp\left(\frac{-t}{\tau_2}\right)\right]$$

Supplementary Note 3 addresses the consequences of competing reactions in the presence of dissolved O_2.

Computational methodology Geometry optimization and harmonic frequency calculations are carried out with the ωB97xD functional and 6-311++G** basis set using Gaussian09.[5] The neutral and ionic species are computed for PHEN, ANTHRA, and DCB, both for the isolated molecules as well as applying the polarization continuum model in acetonitrile as solvent. The vertical excitation energies of the first five excited-singlet states and the first two excited-triplet states for PHEN and ANTHRA are calculated at the CAM-B3LYP / 6-311++G** level of theory. Pertaining to the decarboxylation reaction, the ground-state geometries of neutral CHCA and the corresponding anion are calculated at the ωB97xD / 6-311++G** level of theory. We also compute the minimum energy structures and optimize the frequencies for both cyclohexanecarboxyl (CHC) and cyclohexyl (CH) radicals.

A potential energy surface scan of the decarboxylation reaction is carried out to compute the free energy of the transition state. This is done by varying the R-COO bond distance of the CHC radical in steps of 0.02 $\AA$. The bond angles and dihedral angles related to the separating carbon atoms and the leaving group (CO_2) are fully relaxed. All other internal coordinates are held fixed at the equilibrium geometry values for the CHC radical. The computational results are shown in Supplementary Figures 5 and 8.

Supplementary Note 1: Time-Resolved Electronic Spectroscopy (TRES) Results

Supplementary Figure 1(a) shows the electronic transient absorption spectrum of a 10 mM solution of PHEN in ACN, partitioned over three representative time-windows (dotted blue line, 20-100 fs, dash-dotted green line, 10-20 ps, and solid red line, 800-1300 ps) in Supplementary Figure 1(b). The solid black line in Supplementary Figure 1(b) measured at negative time delays serves as a baseline reference. At 256 nm, an $S_0 \rightarrow S_4$ transition occurs according to our calculations as well as literature reports.[6] The initially populated singlet state (S_4, $\pi\pi^*$), characterized by broad absorption bands at 400 nm and 585 nm, undergoes very fast internal conversion ($\approx$230 fs) to the lowest excited singlet state (S_1). This number is arrived at from a global-fitting of the dataset where the instrument response (150 fs) is deconvolved from the chirp-corrected spectra.

A large fraction of the singlet state population undergoes intersystem crossing to the triplet state, the lifetime of which is beyond the temporal range of the transient absorption experiment.[7,8] Weak vibronic structure is recognizable in the spectrum measured at long time delays, as expected from the vibronic structure measured in the ground-state absorption spectrum (Supplementary Figure 4). The transient absorption observed between 395-500 nm is tentatively assigned to triplet state absorption from reported flash photolysis experiments of PHEN in acetonitrile.[8] Peaks observed between 500 and 600 nm are tentatively assigned to S_1 state absorption from the low quantum yield (0.13) and long fluorescence lifetime (55 ns) of PHEN in cyclohexane (since essential spectroscopic properties of PHEN are remarkably insensitive to solvent polarity).[9]

Upon successive addition of DCB (Supplementary Figure 1c), changes in the spectrum at long time delays can be identified as a decrease in the amplitude of spectral features greater than 450 nm, and the rise of new peaks at 348, 403, and 431 nm. These peaks are mainly assigned to the DCB anion,[10,11] with a minor underlying contribution to the stronger peaks (403 and 431 nm) from the PHEN cation.[6,8,12,13] A control experiment using anthracene is used to corroborate the assignment (Supplementary Figure 2). We find that upon single electron transfer from PHEN* to DCB, the amplitude of the early time spectrum is hardly affected ($\leq 8\%$) whereas the late-time spectrum decreases in amplitude by $\geq 40\%$ (see difference spectrum in Supplementary Figure 2d). Thus, photoinduced bimolecular electron transfer (step 2 in Figure 1, main text), is mediated by diffusion and not fast enough to compete with internal conversion or intersystem crossing in PHEN. The first SET occurs predominantly from the triplet state of PHEN, and perhaps also from S_1 due to the slow ground state recovery.[7,14]

Supplementary Figure 2(a,b) shows the transient electronic absorption spectrum of anthracene photoexcited at 375 nm. This wavelength corresponds to an $S_0 \rightarrow S_1$ transition.[8] The fluorescence

lifetime of anthracene is short (about 5 ns)[15] and the triplet quantum yield is high due to facile singlet fission.[16] The bands observed at 368, 387, 407, 414, 560, 598 nm after photoexcitation are all attributed to singlet state absorption.[6,17] The band appearing at 420 nm at longer time delays is attributed to the formation of a triplet state due to intersystem crossing.[17] Addition of DCB to the solution shows subtle changes in the transient absorption spectrum measured at long time delays (800-1330 ps, Supplementary Figure 2(c)). A difference spectrum shows peaks at 349 and 431 nm, also observed in the case of PHEN-DCB. Thus, a single electron transfer also occurs from the excited state of anthracene to DCB in solution. These peaks, common to the spectra of both arene-DCB systems, likely correspond to the DCB anion.

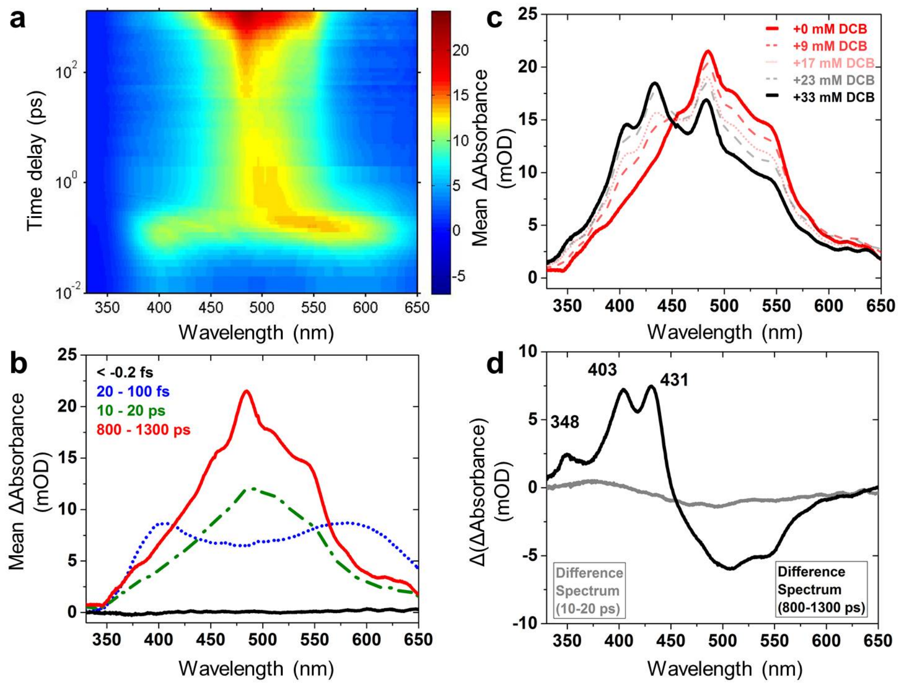

Supplementary Figure 1: Time-resolved electronic spectra of phenanthrene photoexcited at 256 nm. (a) 2D false color map of the time-resolved electronic spectrum (TRES, same as transient electronic absorption spectrum) of PHEN (10 mM) in acetonitrile at 256 nm. Note the logarithmic scale of the time axis. Amplitude units are in mOD, as annotated by the color bar on the right. The spectrum measured at negative times is subtracted for baseline correction. (b) Transient absorption spectra evolving over representative time windows (solid black line is for negative time delays, dotted blue line for 20-100 fs, dash-dotted green line for 10-20 ps, and solid red line for 800-1300 ps). (c) Transient absorption spectra of PHEN at 256 nm measured between 800-1300 ps for increasing concentration (red to black) of DCB. (d) Difference spectra of PHEN in acetonitrile and PHEN + DCB (33 mM) in acetonitrile measured between 10-20 ps (gray) and 800-1300 ps (black).

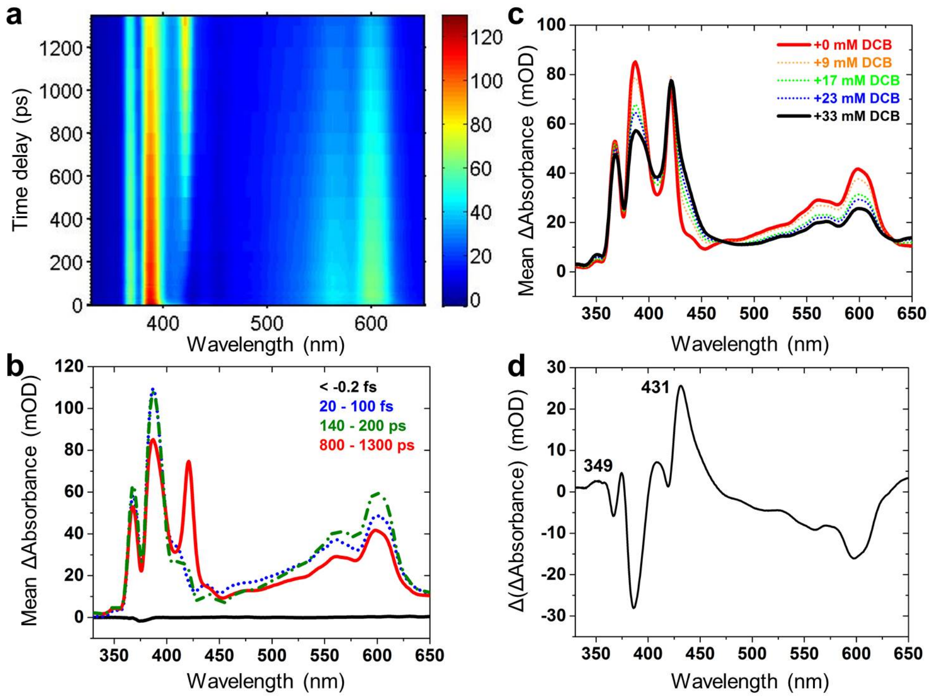

Supplementary Figure 2: Time-resolved electronic spectra of anthracene photoexcited at 375 nm. (a) 2D false color map of the transient electronic spectrum of ANTHRA (10mM) in acetonitrile at 375 nm. Note the linear scale of the time axis. Amplitude units are in mOD, as annotated by the color bar on the right. The spectrum measured at negative times is subtracted for baseline correction. (b) Transient absorption spectra evolving over representative time-windows (solid black line is for negative time delays, dotted blue line for 20-100 fs, dash-dotted green line for 10-20 ps, and solid red line for 800-1300 ps). (c) Transient absorption spectra of ANTHRA at 375 nm measured between 800-1300 ps for increasing concentration (red to black) of DCB. (d) Difference spectra of ANTHRA in acetonitrile and ANTHRA + DCB (33 mm) in acetonitrile measured between 800-1300 ps.

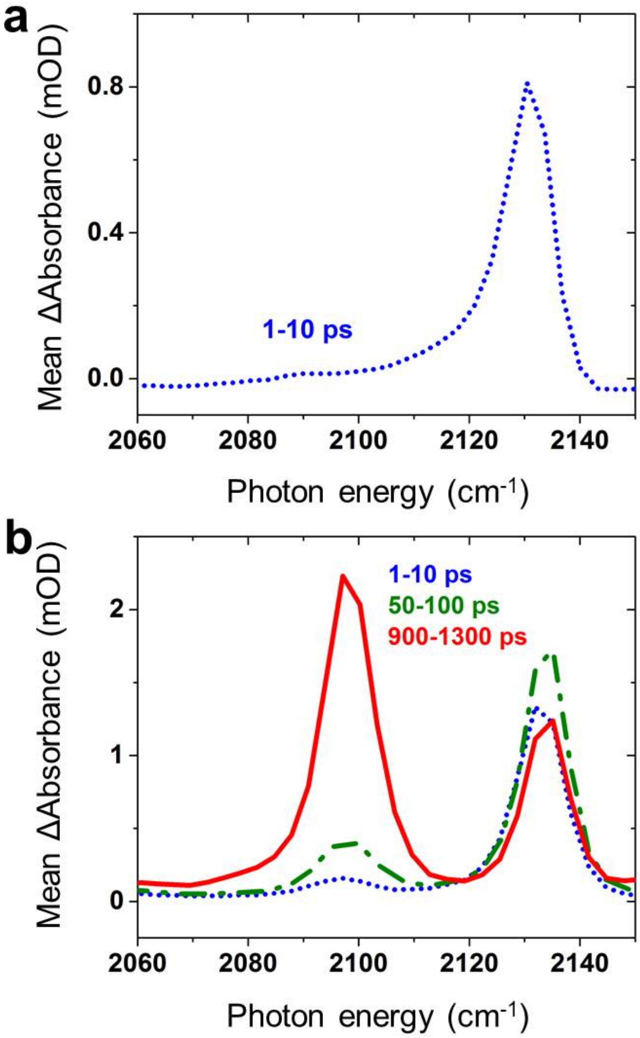

Supplementary Figure 3: Time-resolved infrared spectra of 1,4-dicyanobenzene photoexcited at 280 nm. (a) TRIR spectrum of DCB (25 mM) in ACN-d_3 at 280 nm shows a peak due to the nitrile stretching frequency in the excited state (2135 cm^{-1}) of DCB. (b) TRIR spectra of DCB (25 mM) and PHEN (2.5 mM) in ACN-d_3 at 280 nm show the same peak at early time delays and the nitrile stretching frequency of DCB^- at late time delays (2098 cm^{-1}) from a photoinduced electron transfer.

Supplementary Note 2: UV-VIS Static Absorption Measurements

Supplementary Figure 4(a) shows the UV-VIS absorption spectrum of PHEN, DCB, and CHCA in acetonitrile. To achieve selective excitation of PHEN despite significant overlap of the spectral features of the donor-acceptor pair, we excite the co-oxidant system at 256 nm, close to an absorption minimum of DCB. For an equimolar ratio of the two components, this achieves a discrimination ratio of $\approx$16:1 for preferential excitation of PHEN over DCB. Comparison of the infrared transient absorption spectra measured for excitation at 256 nm and 280 nm indeed confirms selective PHEN $\rightarrow$ PHEN* excitation at the former wavelength from the absence of the nitrile stretch peak of DCB* (2135 cm^{-1}). We note that for excitation wavelengths longer than 280 nm, the photoexcitation of the electron acceptor (DCB $\rightarrow$ DCB*) is likely to be competitive (Supplementary Figure 3) but less likely to play a role in a back electron transfer reaction to the radical due to the short lifetime of DCB^{-} in solution.[18]

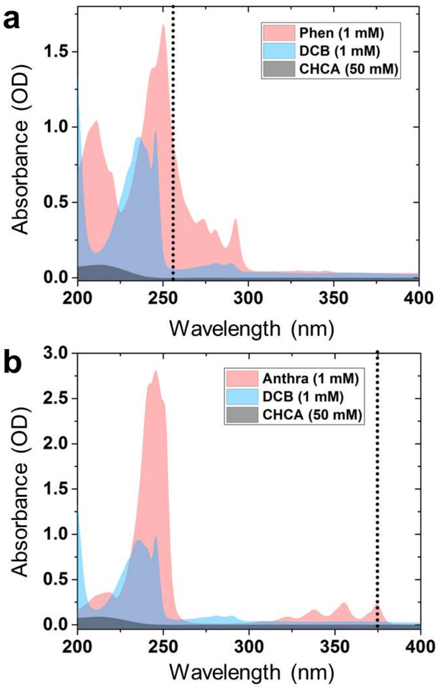

Supplementary Figure 4: Steady-state electronic absorption spectra. (a) UV-Visible absorption spectra of PHEN, DCB, and CHCA in acetonitrile; each solution is measured in a Harrick cell of 200 micron path length. The inset shows the concentration of the solutions used. A black dotted line is used to indicate the choice of the excitation wavelength (256 nm) for the transient absorption studies of this system to order to achieve selective excitation of PHEN in the reaction mixture. (b) UV-Visible absorption spectra of ANTHRA, DCB, and CHCA in acetonitrile. The excitation wavelength for the transient absorption studies of this reaction mixture is 375 nm.

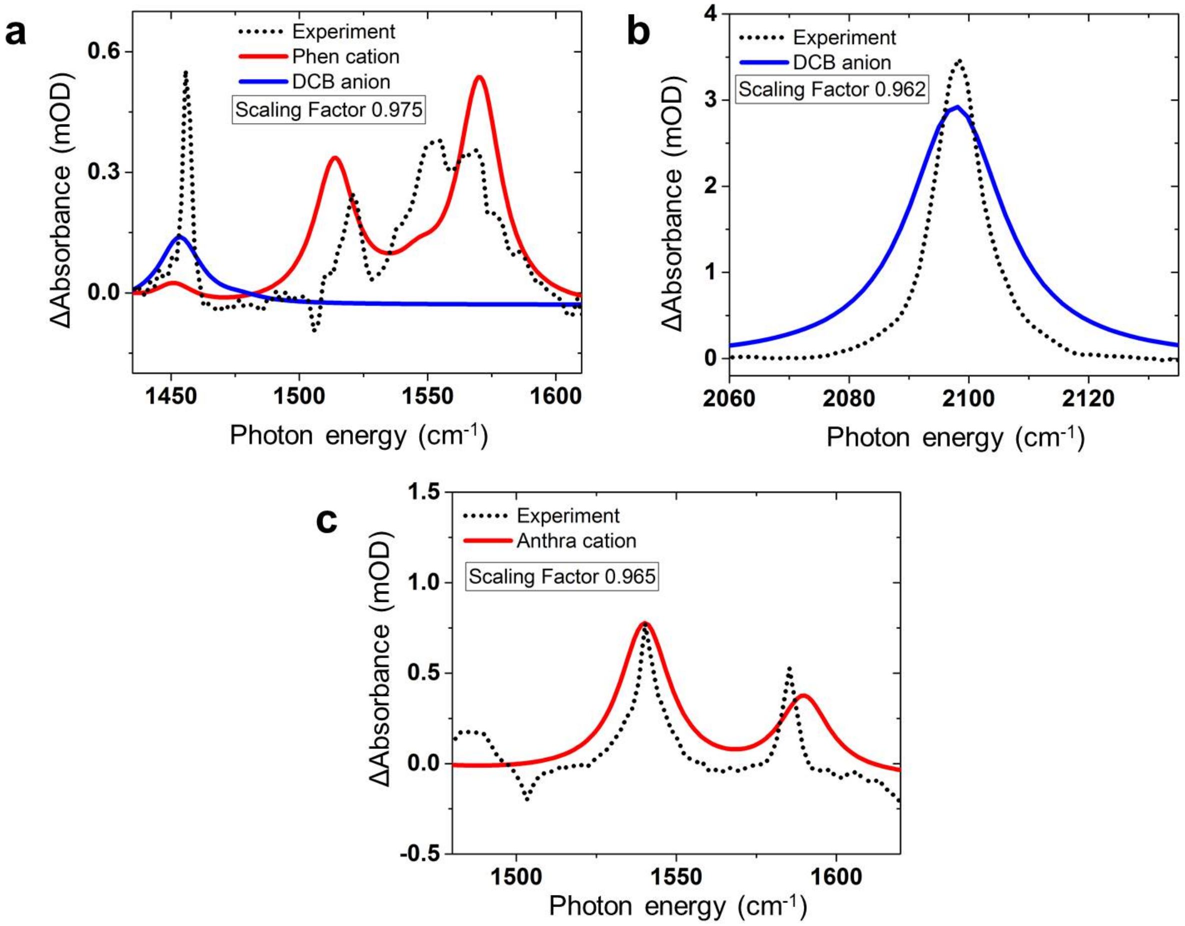

Supplementary Figure 5: Comparison of the computed vibrational spectra with the experiment. Computed IR spectra (ωB97xD / 6-311++G**) of PHEN cation, DCB anion and ANTHRA cation with (a, b) the experimentally measured spectra of PHEN-DCB in ACN-d_3 and (c) ANTHRA-DCB in ACN-d_3. The scaling factors are shown in the inset. The dotted black line in all the panels corresponds to the experiment (see Figure 2 and Supplementary Figure 6 for the false 2D color maps). Solid lines correspond to the theory (red is for cation and blue denotes the anion) to which a Lorentzian broadening of 10-20 cm^{-1} is applied.

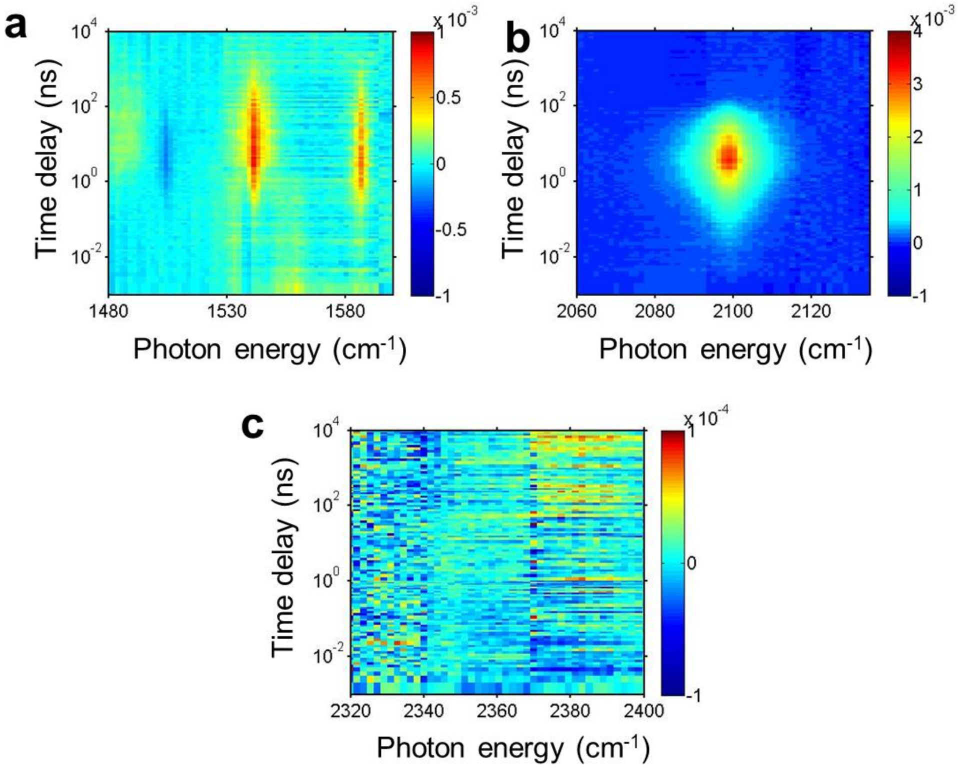

Supplementary Figure 6: Control experiment on photocatalytic decarboxylation reaction using anthracene instead of phenanthrene. (a, b) 2D false color maps of the TRIR spectra of a mixture of ANTHRA (8 mM) and DCB (26 mM) in ACN-d_3, which is photoexcited at 375 nm. The TRIR spectra are measured in the regions of the anthracene in-plane ring distortion and nitrile stretch modes. Note the logarithmic scale of the time axis. Amplitude units, measured in optical density (OD), are annotated by the color bar on the right. (c) False color map of the TRIR spectrum of the full reaction mixture - ANTHRA (4mM):DCB:CHCA:NaOH = 1:3:6:6 - photoexcited at 375 nm and measured in the region of the antisymmetric stretch of CO_2. No evidence for oxidative decarboxylation is seen in this experiment.

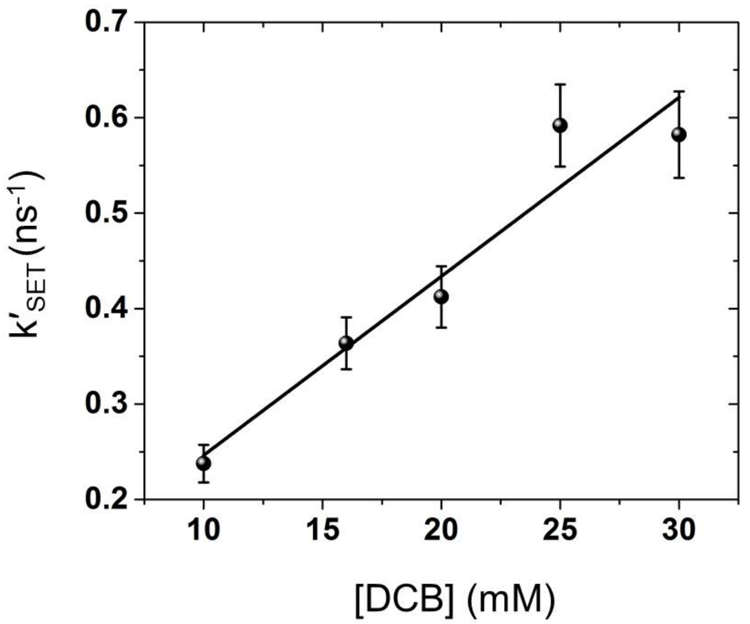

Supplementary Figure 7: Diffusion-limited photoinduced electron transfer. Pseudo-first order kinetic plot for the photoinduced single electron transfer (PHEN* to DCB) rate constant as a function of the concentration of DCB. The error bars denote one standard error in the fitting procedure used to obtain the rate constants.

Supplementary Note 3: Lifetime Extraction of the Reaction Intermediate in Presence of $PHEN^+$ Quenching by Superoxide

The possible loss mechanisms for $PHEN^+$, reactive and non-reactive (with respect to the decarboxylation reaction), include:

$(1) PHEN^+ + O_2^- \xrightarrow{k_{(1)}} PHEN + O_2 ....[NonReactive]$

$(2) PHEN^+ + DCB^- \xrightarrow{k_{(2)}} PHEN + DCB ....[NonReactive]$

$(3) PHEN^+ + OH^- \xrightarrow{k_{(3)}} PHEN + {}^{\bullet}OH ....[NonReactive]$

$(4) PHEN^+ + RCOO^- \xrightarrow{k_{(4)}} PHEN + RCOO^{\bullet} ....[Reactive]$

It must be noted that reactions (3) and (4) occur exclusively in the presence of CHCA and NaOH in solution. The serial numbering of these reactions (1)-(4) should not be confused with steps 1-4 introduced in Figure 1 of the main paper. To distinguish between these representations, we use parentheses (1)-(4) in our discussion here pertaining to the possible loss mechanisms for $PHEN^+$. In the absence of CHCA + NaOH, we have the rate of decay of $PHEN^+$,

$$-\frac{d[PHEN^+]}{dt}\bigg|_{(1)-(2)} = k_{(1)}[PHEN^+][O_2^-] + k_{(2)}[PHEN^+][DCB^-] ....(i)$$

In the presence of CHCA + NaOH, we have

$$-\frac{d[PHEN^+]}{dt}\bigg|_{(1)-(4)} = k_{(1)}[PHEN^+][O_2^-] + k_{(2)}[PHEN^+][DCB^-] + k_{(3)}[PHEN^+][OH^-] + k_{(4)}[PHEN^+][RCOO^-] ....(ii)$$

Under the conditions of the photocatalytic decarboxylation reaction,

$$[RCOO^-]; [OH^-] >> [PHEN^+]; [DCB^-]; [O_2^-]$$

because the latter set of species is formed due to electron transfer after photoexcitation of PHEN. We can assume that $[RCOO^-]$ and $[OH^-]$ do not change appreciably during the course of the reaction. Eqn. (ii) therefore simplifies to

$$-\frac{d[PHEN^+]}{dt}\bigg|_{(1)-(4)} = k_{(1)}[PHEN^+][O_2^-] + k_{(2)}[PHEN^+][DCB^-] + \left(k'_{(3)} + k'_{(4)}\right)[PHEN^+] ....(iii)$$

Here, $k'_{(3)} = k_{(3)}[OH^-]$ and $k'_{(3)} = k_{(3)}[RCOO^-]$ are the pseudo first-order reaction coefficients for reactions (3) and (4), respectively.

The difference between the rates of decay of $PHEN^+$ with and without the presence of CHCA + NaOH, [(iii) – (i)], is

$$-\frac{d[PHEN^+]}{dt}\bigg|_{(1)-(4)} + \frac{d[PHEN^+]}{dt}\bigg|_{(1)-(2)} \approx \left(k'_{(3)} + k'_{(4)}\right)[PHEN^+]$$

Experimentally, we find this difference to be approximately 100 ns (*e.g.*, the rates are 230 $\pm$ 70 ns and 340 $\pm$ 70 ns, respectively, with and without CHCA + NaOH). We use the faster decay rate in the kinetic model because the fate of the decarboxylation step is determined by the overall decay of $PHEN^+$. The lifetime of the radical (τ_2 = 1/k_2) is solely determined by the risetime of CO_2- the mean and standard deviation over nine such measurements are used to evaluate the lifetime of the carboxyl radical (500 ns) and the error bar, respectively ($\pm$120 ns).

The non-reactive decay channels of $PHEN^+$ somewhat limit the success of the decarboxylation step in our experiment. Accordingly, the estimated appearance time of the radical is determined by the overall decay of $PHEN^+$ induced by both slower reactive and faster non-reactive channels. The time-window over which the radical is populated is likely to be extended to microseconds if O_2 is carefully removed and / or back electron transfer from DCB^- is inhibited by adding water for the efficient solvation and separation of the ion pair. Notwithstanding a precise knowledge of the O_2 concentration in the reaction medium, the kinetic model presented in Figure 4 is robust and can be formally applied to estimate the lifetimes of the reactive intermediates under different reaction conditions.

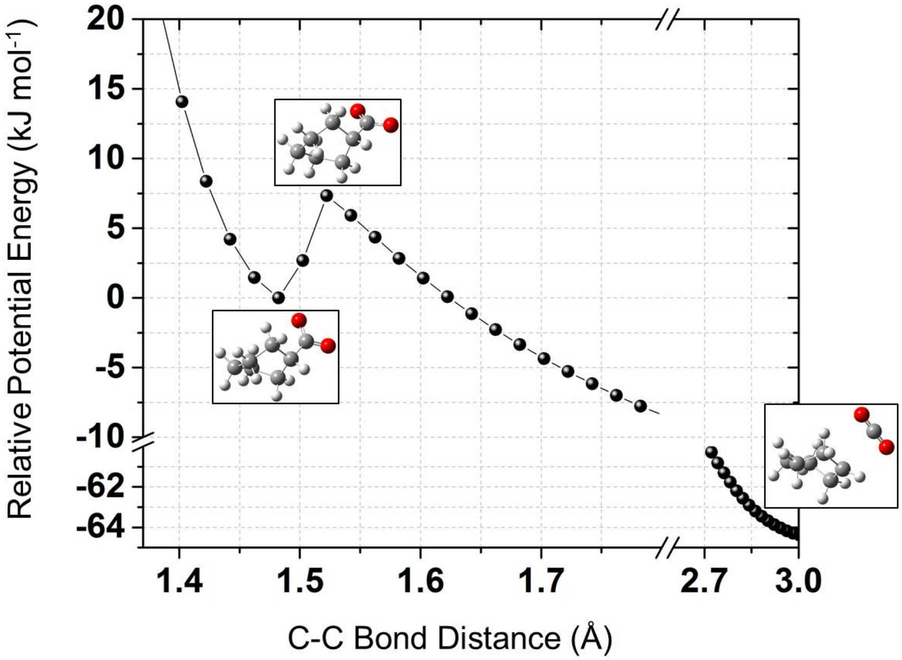

Supplementary Figure 8: Activation controlled decarboxylation. Relaxed potential energy scan (ωB97xD / 6-311++G**) for the decarboxylation of the cyclohexanecarboxyl radical shows a saddle point corresponding to the transition state.

Supplementary Note 4: Direct Observation of the Carboxyl Radical as a Reaction Intermediate using TRIR Spectroscopy

Photocatalytic decarboxylation of CHCA in an alkaline solution is studied at a higher power of 256 nm (800 nJ) at the Ultra Facility at RAL (Supplementary Figure 9).[19] This instrument operates at 10 kHz and provides up to a 500 cm^{-1} spectral coverage in a single experiment. It also provides higher power output at the desired pump wavelength in comparison to the LIFEtime facility. The pump repetition rate is set at 500 Hz. The concentrations of the reactants in this experiment are same as Figure 3 of the paper. However, the experiment is carried out in a closed-flow cell to minimize oxygen contamination, after purging the solvent with dry N_2 for over thirty minutes. Post purging, the whole flow system is flushed with nitrogen and the head space of the bottle is filled with dry N_2 and sealed. Under better purged (O_2 free) conditions, we find that the time constants for the decays of $PHEN^+$ and DCB^- become comparable ($\approx$ 200-300 ns). The kinetics suggest that back electron transfer is mainly diffusive, in the absence of any fast components otherwise indicative of recombination of the geminate ion pair. Note that better oxygen removal in this experiment prolongs the time window over which the carboxyl radical is observed in comparison to estimates obtained from Figure 4 of the same experiment under different conditions using the LIFEtime facility.

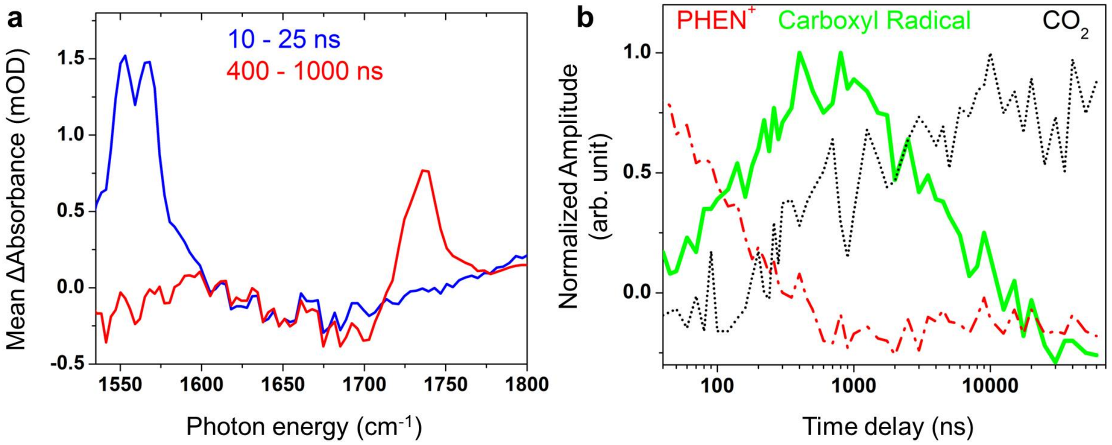

Supplementary Figure 9: Time-resolved infrared spectra at high pump fluence. (a) TRIR spectra measured at 800 nJ UV (256 nm) pump power. $PHEN^+$ absorption bands appear in tens of nanoseconds (blue trace) and the asymmetric carboxyl stretch of the radical at 1735 cm^{-1} appears over hundreds of nanoseconds (red trace) (b) Normalized peak amplitudes for $PHEN^+$ (red trace), carboxyl radical (green trace) and CO_2 (black trace) infrared absorption bands as a function of the pump-probe time delay reveal that the cyclohexanecarboxyl radical is indeed a reaction intermediate.

Supplementary References

1. Greetham, G. M. *et al.* A 100 kHz time-resolved multiple-probe femtosecond to second infrared absorption spectrometer. *Appl. Spectrosc.* **70**, 645–653 (2016).

2. Roberts, G. M., Marroux, H. J. B., Grubb, M. P., Ashfold, M. N. R. & Orr-Ewing, A. J. On the participation of photoinduced N-H bond fission in aqueous adenine at 266 and 220 nm: A combined ultrafast transient electronic and vibrational absorption spectroscopy study. *J. Phys. Chem. A* **118**, 11211–11225 (2014).

3. Yoshimi, Y., Itou, T. & Hatanaka, M. Decarboxylative reduction of free aliphatic carboxylic acids by photogenerated cation radical. *Chem. Commun.* 5244–5246 (2007).

4. Steinfeld, J. I., Francisco, J. S. & Hase, W. L. *Chemical Kinetics and Dynamics*, vol. 3 (Prentice Hall Englewood Cliffs, New Jersey, 1989).

5. Frisch, M. J. *et al.* Gaussian 09, revision d.01. *Gaussian, Inc., Wallingford CT* (2013).

6. Salama, F., Joblin, C. & Allamandola, L. J. Electronic absorption-spectroscopy of matrix-isolated polycyclic aromatic hydrocarbon cations. 2. The phenanthrene cation ($C_{14}H_{10}{}^{+}$) and its 1-methyl derivative. *J. Chem. Phys.* **101**, 10252–10262 (1994).

7. Powell, G. L. Kinetics of excited singlet and triplet states of vapor-phase phenanthrene. *J. Chem. Phys.* **47**, 95–101 (1967).

8. Grellmann, K. H., Watkins, A. R. & Weller, A. Electron-transfer mechanism of fluorescence quenching in polar solvents. 1. Dicyanobenzene as quencher. *J. Phys. Chem.* **76**, 469–473 (1972).

9. Zhang, P., Winnik, M. A. & Wang, Z. Y. The spectroscopy and photophysics of 3 9,10-diaryl phenanthrene derivatives. *J. Photochem. Photobiol. A* **89**, 13–17 (1995).

10. Robinson, E. A. & Schulte-Frohlinde, D. Pulse radiolysis of 1,4-dicyanobenzene in aqueous solutions in the presence and absence of thallium(1) ions. *J. Chem. Soc. Faraday Trans.* **69**, 707–718 (1973).

11. Grellmann, K. H., Watkins, A. R. & Weller, A. Electron-transfer mechanism of fluorescence quenching in polar solvents. 2. tetracyanoethylene and tetracyanobenzene as quenchers. *J. Phys. Chem.* **76**, 3132–3137 (1972).

12. Khan, Z. H. Electronic-spectra of radical cations and their correlation with photoelectron-spectra .6. a reinvestigation of 2-ring, 3-ring, and 4-ring condensed aromatics. *Acta Phys. Pol. A* **82**, 937–955 (1992).

13. Hiratsuka, H., Hatano, Y., Tanizaki, Y. & Mori, Y. Polarized absorption-spectra of aromatic radicals in stretched polymer-films .6. radical ions of some aromatic-molecules. *J. Chem. Soc. Faraday Trans.* **81**, 1653–1664 (1985).

14. Taen, S. & Gondo, Y. Nonexponential phosphorescence decay of phenanthrene in biphenyl. *Chem. Phys. Lett.* **123**, 441–444 (1986).

15. Boens, N. *et al.* Fluorescence lifetime standards for time and frequency domain fluorescence spectroscopy. *Anal. Chem.* **79**, 2137–2149 (2007).

16. Bowers, P. G. & Porter, G. Triplet state quantum yields for some aromatic hydorcarbons and xanthene dyes in dilute solution. *Proc. Royal Soc. A* **299**, 238–258 (1967).

17. Lang, B. *et al.* Broadband ultraviolet-visible transient absorption spectroscopy in the nanosecond to microsecond time domain with sub-nanosecond time resolution. *Rev. Sci. Instrum.* **84**, 073107 (2013).

18. Yoshimi, Y. *et al.* Radical addition to acrylonitrile via catalytic photochemical decarboxylation of aliphatic carboxylic acids. *Tetrahedron Lett.* **54**, 4324–4326 (2013).

19. Greetham, G. M. *et al.* Ultra: A unique instrument for time-resolved spectroscopy. *Appl. Spectrosc.* **64**, 1311–1319 (2010).
